# Serum metabolomic profiles in BALB/c mice induced by *Babesia microti* infection

**DOI:** 10.3389/fcimb.2023.1179967

**Published:** 2023-04-28

**Authors:** Liang Shen, Chunhua Wang, Ruilin Wang, Xue Hu, Shiying Liao, Wentong Liu, Aoling Du, Shengwei Ji, Eloiza May Galon, Hang Li, Xuenan Xuan, Juan Xiao, Mingming Liu

**Affiliations:** ^1^ Central Laboratory, Xiangyang Central Hospital, Affiliated Hospital of Hubei University of Arts and Science, Xiangyang, China; ^2^ School of Basic Medicine, Hubei University of Arts and Science, Xiangyang, China; ^3^ National Research Center for Protozoan Diseases, Obihiro University of Agriculture and Veterinary Medicine, Obihiro, Japan

**Keywords:** *Babesia microti*, mouse model, serum, metabolome, LC−MS

## Abstract

**Introduction:**

The protozoan parasite *Babesia microti* is the primary cause of human babesiosis. This parasite invades and multiplies inside red blood cells (RBCs), and infections differ significantly based on the age and immune competency of the host. The aim of this study was to investigate the use of serum metabolic profiling to identify systemic metabolic variations between *B. microti*-infected mice and noninfected controls.

**Methods:**

A serum metabolomics analysis of BALB/c mice that had been intraperitoneally injected with 10^7^ *B. microti*-infected RBCs was performed. Serum samples from the early infected group (2 days postinfection), the acutely infected group (9 days postinfection), and the noninfected group were collected and evaluated using a liquid chromatography−mass spectrometry (LC−MS) platform. Principal component analysis (PCA), partial least squares discriminant analysis (PLS-DA), and orthogonal partial least squares discriminant analysis (OPLS-DA) identified metabolomic profiles that differentiated the *B. microti*-infected and noninfected groups.

**Results:**

Our results confirm that the serum metabolome is significantly influenced by acute *B. microti* infection and show that infection results in dysregulation of metabolic pathways and perturbation of metabolites. Acutely infected mice displayed perturbations in metabolites associated with taurine and hypotaurine metabolism, histidine metabolism, and arachidonic acid metabolism. Taurocholic acid, anserine, and arachidonic acid may be potential candidates as serological biomarkers for diagnosing *B. microti* infection at the acute stage. These metabolites could be further examined for their role in disease complexity.

**Discussion:**

Our findings demonstrate that the acute stage of *B. microti* infection induces abnormalities in the metabolites present in mouse serum and provide new insight into the mechanisms involved in systemic metabolic changes that occur during *B. microti* infection.

## Introduction

*Babesia microti* is the most common intraerythrocytic parasite that causes human babesiosis (Vannier et al., 2015). This parasite is mainly transmitted by tick bites, but it has been shown that it can also spread through blood transfusion, and a few reports have suggested that it may be transmitted to a developing fetus via the placenta (Tufts and Diuk-Wasser, 2021). Severe symptoms in neonates or older adults could be related to depressed cellular immunity in immunocompromised individuals, particularly splenectomized individuals (Yi et al., 2018). Death occurs in 10% of patients who are hospitalized for severe *B. microti* infection (Bloch et al., 2022). The mild disease caused by *B. microti* typically manifests as intermittent fever, malaise, and weakness (Vannier and Krause, 2012).

Metabolomics, a high-throughput analytical profiling technique in which the small biochemical compounds produced during metabolic processes are measured and compared, offers an advantage for identifying and quantifying a wide range of minor chemical metabolites, evaluating disease development, and determining physiological status based on analysis of complex biological samples (Chen et al., 2022). This approach facilitates the exploration of various metabolic changes that occur throughout the parasite life cycle and fulfils the demand for advanced diagnostics and precision therapeutics (Yu et al., 2021). Moreover, it can be used to diagnose disease, screen for biomarkers, and explore the mechanisms of pathogenesis (Gowda et al., 2008).

In recent years, metabolomics analysis has been extensively utilized in the study of protozoan parasites, including *Cryptosporidium baileyi*, *Plasmodium berghei*, *Toxoplasma gondii*, and *Trypanosoma cruzi* (Lizardo et al., 2019; Zhou et al., 2019; Hajialiani et al., 2021; Wu et al., 2021). A metabolomics study found that human malarial infections are associated with the presence of elevated amounts of bile acids, bile pigments, and steroid hormones. Upregulated levels of metabolites such as histidine, ornithine, pantothenate, phenylalanine, pipecolate and valine were observed in individuals with severe malaria (Das et al., 2022). In addition, serum metabolic analysis of mice infected with *T. gondii* revealed changes in glycerophospholipid metabolism, amino acid biosynthesis, and tyrosine metabolism (Zhou et al., 2018).

Compared to *in vitro* studies of parasite biology, serum metabolomic analysis provides a potent tool for the discovery of clinical biomarkers (Lakshmanan et al., 2012). Aiming to gain a better understanding of *B. microti* pathogenesis and identify new biomarkers that possess potential diagnostic or therapeutic value, we analyzed the serum metabolic profiles of *B. microti*-infected mice using an untargeted liquid chromatography−mass spectrometry (LC−MS) approach. We successfully identified alterations in several specific metabolic pathways and in specific metabolites, as well as potential diagnostic and therapeutic targets, in serum obtained from mice with *B. microti* infection.

## Materials and methods

### Animals and parasites

Female BALB/c mice 6–9 weeks old (China Three Gorges University) were used in this study. The Peabody mjr strain of *B. microti* (ATCC^®^ PRA-99™) was intraperitoneally administered to BALB/c mice with 10^7^ parasites per animal when parasitemia was 20–30% in the donor mice.

### Sample preparation

Blood samples from the early infected group (2 days postinfection), the acutely infected group (9 days postinfection), and the noninfected group were prepared as previously described (Wikoff et al., 2009). The blood samples were collected through an intracardiac bleed into microcentrifuge tubes and allowed to clot at room temperature for 3 h, followed by centrifugation at 3,000 × g for 10 min at 4°C. The serum was immediately frozen in liquid nitrogen and subsequently stored at −80 ° until use.

### Extraction of serum metabolites

Frozen serum samples were removed from the −80°C freezer, thawed, and vortexed for 10 sec. A total of 50 μL of the sample was mixed with 300 μL of 20% acetonitrile/methanol internal standard extractant. The mixture was thoroughly vortexed and centrifuged at 8,000 × g for 10 min at 4°C. Then, 200 μL of the supernatant was placed at −20°C for 30 min, followed by centrifugation at 8,000 × g for 3 min at 4°C. Approximately 180 μL of the supernatant was used in the analysis.

### Untargeted LC−MS metabolomics analysis

LC−MS metabolomics analysis was performed at Metware Biotechnology Co., Ltd. (Wuhan, China). The analytical parameters were set as follows. Ultra-performance liquid chromatography (UPLC) was performed on a Waters ACQUITY UPLC HSS T3 C18 column (1.8 µm, 2.1 mm × 100 mm) at a column temperature of 40°C, a flow rate of 0.4 mL/min, and an injection volume of 2 μL. The solvent system was water (0.1% formic acid): acetonitrile (0.1% formic acid); the gradient program was 95:5 V/V at 0 min, 10:90 V/V at 11.0 min, 10:90 V/V at 12.0 min, 95:5 V/V at 12.1 min, 95:5 V/V at 14.0 min. Quality control (QC) samples were used with each serum sample to evaluate the reliability and reproducibility of the LC−MS system.

### Data processing and statistical analysis

Serum metabolites were analyzed using LC−MS. The original LC−MS data file was converted to mzML format using ProteoWizard software. The XCMS program was used to perform peak extraction, alignment, and retention time correction. The peak area was corrected using the “SVR” method. Metabolic features detected in at least 50% of the members of any set of samples were retained. The information used to identify specific metabolites was acquired through a search of the self-compiled metabolite database MWDB (Metware Biotechnology Co., Ltd., Wuhan, China) and integration of the results with information obtained from public databases and metDNA.

The criteria used to screen for differential metabolites were as follows: variable importance in projection (VIP) ≥1, fold change ≥2 or ≤0.5 and *p* value < 0.05. VIP values were obtained from the orthogonal partial least squares discriminant analysis (OPLS-DA) results, and *p* values were derived from a two-tailed Student’s t test. The data was log2-transformed and mean-centered before OPLS-DA. To avoid overfitting, a permutation test with 200 permutations was performed.

Graphs showing principal component analysis (PCA), partial least squares discriminant analysis (PLS-DA), OPLS-DA and volcano plots were constructed using R software (Version 4.2.2). The identified metabolites were annotated using the compound Human Metabolome Database (HMDB) (https://hmdb.ca/metabolites) (Wishart et al., 2022), and pathway analysis was performed on the differentially expressed metabolites detected in electrospray ionization (ESI)− and ESI+ modes using MetaboAnalyst 5.0 (https://www.metaboanalyst.ca) (Pang et al., 2022). Significant enrichment of metabolic pathways among a given list of metabolites was determined based on a hypergeometric test’s *p* value. Data analysis of abnormal metabolites was performed using GraphPad Prism 8. The t test was used to determine statistically significant differences between the means of variables. A difference was considered statistically significant when the *p* value was less than 0.05.

## Results

### Collection of serum samples from mice

**Figure 1:**
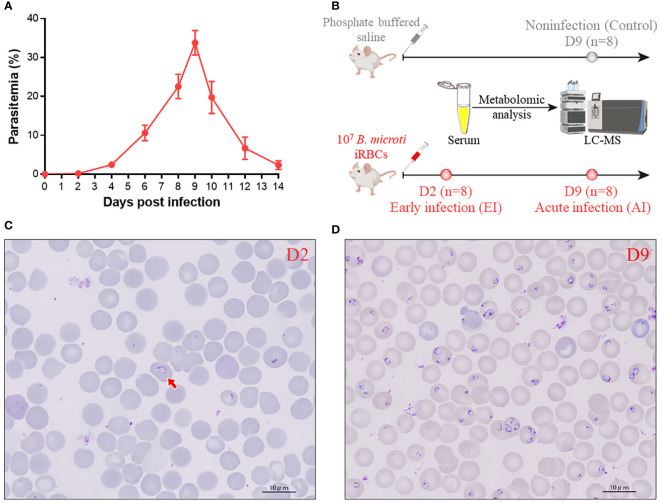
Data on the samples used in this study. **(A)** Percentage of peripheral blood erythrocytes infected with *B. microti* over time. **(B)** Schematic showing how serum samples were prepared for LC−MS metabolomics analysis. **(C, D)** Parasite images at the early stage (2 days postinfection) **(C)** and at the acute stage (9 days postinfection) **(D)** Arrow: iRBC.

Parasitemia was detectable 2 days postinfection; it increased rapidly to a peak at Day 9 (33.79%) and subsequently decreased as a result of the immune response (**Figure 1A**). The serum samples obtained from the infected group on Day 2 and Day 9 and those obtained from the noninfected group on Day 9 were subjected to metabolomics analysis by LC−MS (**Figure 1B**). Images of the parasites present in mice with early and acute infections are shown in **Figures 1C, D**, respectively.

### Identification of serum metabolites

**Figure 2:**
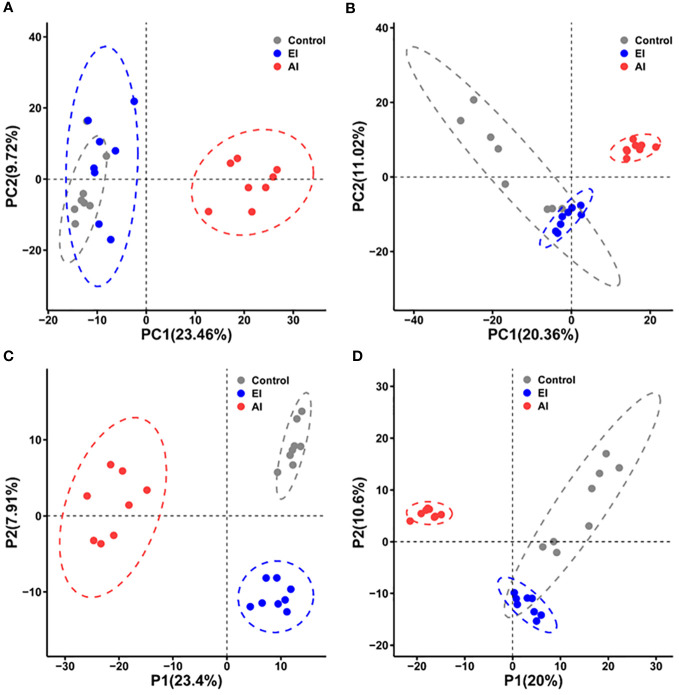
Differential metabolic profiles of mouse serum during *B. microti* infection. **(A, B)** Principal component analysis (PCA) score scatter plots of metabolites obtained through LC−MS in ESI− mode **(A)** and ESI+ mode **(B)**. **(C, D)** Partial least squares discriminant analysis (PLS-DA) separation of the metabolites presents in the three groups in ESI− mode **(C)** and ESI+ mode **(D)**. EI, early infected group; AI, acutely infected group; Ctrl, noninfected group.

Two-dimensional PCA was used to compare the metabolic profiles of the mice in different groups. Despite the fact that the acutely infected group appeared to show a metabolic profile that differed from the profiles of the early infected group and the noninfected group, the PCA score plots obtained under either ESI− mode (**Figure 2A**) or ESI+ mode (**Figure 2B**) did not clearly differentiate the early infected group from the noninfected group. Cross-validated two-dimensional PLS-DA models were used to perform further multivariate analysis, and that analysis distinctly differentiated the three groups of mice in both ESI− mode (**Figure 2C**) and ESI+ mode (**Figure 2D**).

**Figure 3:**
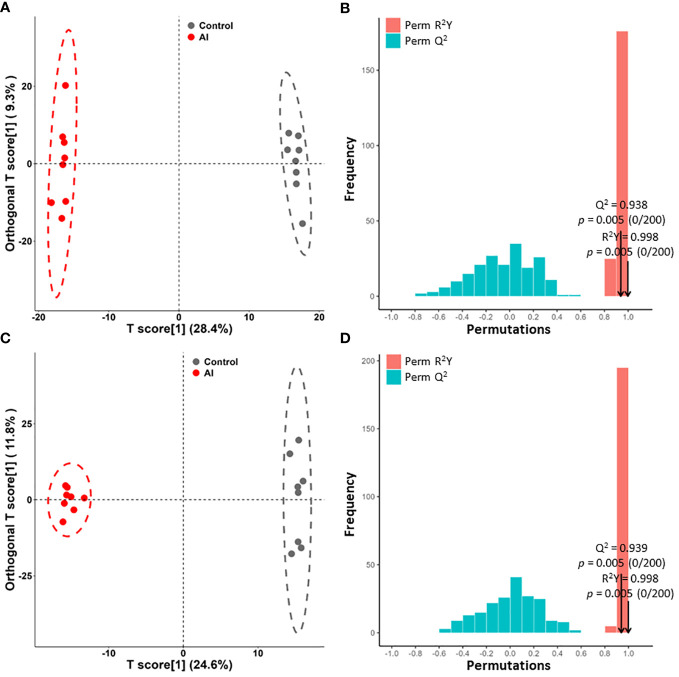
Orthogonal partial least squares discriminant analysis (OPLS-DA) of the metabolite profile. **(A, C)** OPLS-DA score plots between the acutely infected group (AI) and the noninfected group (Ctrl) in ESI− mode **(A)** and ESI+ mode **(C)**. **(B, D)** OPLS-DA model with 200 permutation tests between AI and Ctrl in ESI− mode **(B)** and in ESI+ mode **(D)**.

An OPLS-DA model was used to further analyze the differential metabolites between the acutely infected group and the noninfected group. According to the OPLS-DA scores, the two groups were separated into different regions in both ESI− mode (**Figure 3A**) and ESI+ mode (**Figure 3C**). Comparison of the results of the OPLS-DA model test for the acutely infected group and the noninfected group in ESI− mode (R^2^Y = 0.998, Q^2^ = 0.938, *p* value = 0.005) (**Figure 3B**) and in ESI+ mode (R^2^Y = 0.998, Q^2 ^= 0.939, *p* value = 0.005) (**Figure 3D**) indicated that the OPLS-DA model had a satisfactory fit, with effective stability and predictability. The OPLS-DA model comparing the metabolite profiles of the early infected group and the noninfected group is shown in **Figure S1**. Meanwhile, **Figure S2** shows the OPLS-DA model comparing the metabolite profiles of the early infected group and the acutely infected group.

### Perturbed metabolite identification

**Figure 4:**
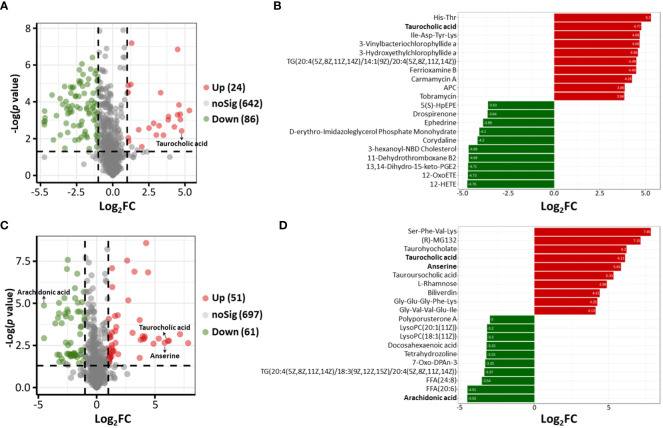
**(A, C)** Volcano plots showing differential metabolites between the acutely infected group (AI) and the noninfected group (Ctrl) in ESI− mode **(A)** and in ESI+ mode **(C)**. Each dot represents a metabolite; the red dots represent significantly upregulated metabolites, and the green dots represent significantly downregulated metabolites. **(B, D)** Linear discriminant analysis (LDA) results showing the top 40 enriched metabolites that were significantly upregulated (red) or downregulated (green) between the AI and Ctrl groups in ESI− mode **(B)** and in ESI+ mode **(D)**.

Based on the results of OPLS-DA, VIP ≥1, fold change ≥2 or ≤0.5 and *p* value < 0.05 were used as thresholds to further confirm the presence of differential metabolites. In ESI− mode, 110 differential metabolites were identified in the acutely infected group versus the noninfected group; these included 24 metabolites that increased significantly and 86 that decreased significantly (**Figure 4A**). In ESI+ mode, 112 differential metabolites were identified in the acutely infected group versus the noninfected group; the levels of 51 of these metabolites increased significantly, and those of 61 decreased significantly (**Figure 4C**). **Figures 4B, D** show the top 20 metabolites identified in the ESI− and ESI+ modes, respectively, using linear discriminant analysis. A list of the top 40 perturbed metabolites identified is shown in **Table 1**.

**Table 1:** List of dysregulated metabolites identified in acutely infected group in ESI− mode and ESI+ mode.

Mode	No	Metabolite	Formula	Mass	*RT (min)*	HMDB
ESI-	1	His-Thr	C_10_H_16_N_4_O_4_	256.11	4.76	HMDB0028895
	2	Taurocholic acid	C_26_H_45_NO_7_S	515.29	5.82	HMDB0000036
	3	Ile-Asp-Tyr-Lys	C_25_H_39_N_5_O_8_	537.28	6.86	–
	4	3-Vinylbacteriochlorophyllide a	C_35_H_36_MgN_4_O_5_	616.25	6.83	–
	5	3-Hydroxyethylchlorophyllide a	C_35_H_36_MgN_4_O_6_	632.25	6.65	–
	6	TG(20:4(5Z,8Z,11Z,14Z)/14:1(9Z)/20:4(5Z,8Z,11Z,14Z))	C_57_H_92_O_6_	872.69	8.87	HMDB0054089
	7	Ferrioxamine B	C_25_H_45_FeN_6_O_8_	613.26	6.85	HMDB0240270
	8	Carmamycin A	C_25_H_45_N_3_O_6_S	515.30	6.85	–
	9	APC	C_33_H_38_N_4_O_8_	618.27	6.85	HMDB0060661
	10	Tobramycin	C_18_H_37_N_5_O_9_	467.26	6.35	HMDB0014822
	11	5(S)-HpEPE	C_20_H_30_O_4_	334.21	8.84	–
	12	Drospirenone	C_24_H_30_O_3_	366.22	9.07	HMDB0015467
	13	Ephedrine	C_10_H_15_NO	165.12	9.07	HMDB0015451
	14	D-erythro-Imidazoleglycerol Phosphate Monohydrate	C_6_H_11_N_2_O_6_P	238.04	3.62	–
	15	Corydaline	C_22_H_27_NO_4_	369.19	9.08	–
	16	3-hexanoyl-NBD Cholesterol	C_39_H_58_N_4_O_5_	662.44	9.16	–
	17	11-Dehydrothromboxane B2	C_20_H_32_O_6_	368.22	9.17	HMDB0004242
	18	13,14-Dihydro-15-keto-PGE2	C_20_H_32_O_5_	352.23	6.68	HMDB0002776
	19	12-OxoETE	C_20_H_30_O_3_	318.22	9.34	HMDB0013633
	20	12-HETE	C_20_H_32_O_3_	320.24	9.16	HMDB0006111
ESI+	21	Ser-Phe-Val-Lys	C_23_H_37_N_5_O_6_	479.27	5.78	–
	22	(R)-MG132	C_26_H_41_N_3_O_5_	475.30	6.81	–
	23	Taurohyocholate	C_26_H_45_NO_7_S	515.29	5.77	HMDB0011637
	24	Taurocholic acid	C_26_H_45_NO_7_S	515.29	5.78	HMDB0000036
	25	Anserine	C_10_H_16_N_4_O_3_	240.12	5.78	HMDB0000194
	26	Tauroursocholic acid	C_26_H_45_NO_7_S	515.29	5.78	HMDB0000889
	27	L-Rhamnose	C_6_H_12_O_5_	164.07	5.94	HMDB0000849
	28	Biliverdin	C_33_H_34_N_4_O_6_	582.25	5.54	HMDB0001008
	29	Gly-Glu-Gly-Phe-Lys	C_24_H_36_N_6_O_8_	536.26	7.37	–
	30	Gly-Val-Val-Glu-Ile	C_23_H_41_N_5_O_8_	515.30	6.81	–
	31	Polyporusterone A	C_28_H_46_O_6_	478.33	10.13	HMDB0038495
	32	LysoPC(20:1(11Z))	C_28_H_56_NO_7_P	549.38	10.02	HMDB0010391
	33	LysoPC(18:1(11Z))	C_26_H_52_NO_7_P	522.36	8.71	HMDB0010385
	34	Docosahexaenoic acid	C_22_H_32_O_2_	328.24	9.29	HMDB0002183
	35	Tetrahydrozoline	C_13_H_16_N_2_	200.13	9.15	–
	36	7-Oxo-DPAn-3	C_22_H_32_O_3_	344.23	9.06	–
	37	TG(20:4(5Z,8Z,11Z,14Z)/18:3(9Z,12Z,15Z)/20:4(5Z,8Z,11Z,14Z))	C_61_H_96_O_6_	924.72	8.40	HMDB0054265
	38	FFA(24:8)	C_24_H_30_O_3_	366.22	9.07	–
	39	FFA(20:6)	C_20_H_28_O_2_	300.21	8.58	–
	40	Arachidonic acid	C_20_H_32_O_2_	304.24	9.05	HMDB0001043

RT, retention time.

### Analysis of dysregulated metabolic pathways

**Figure 5:**
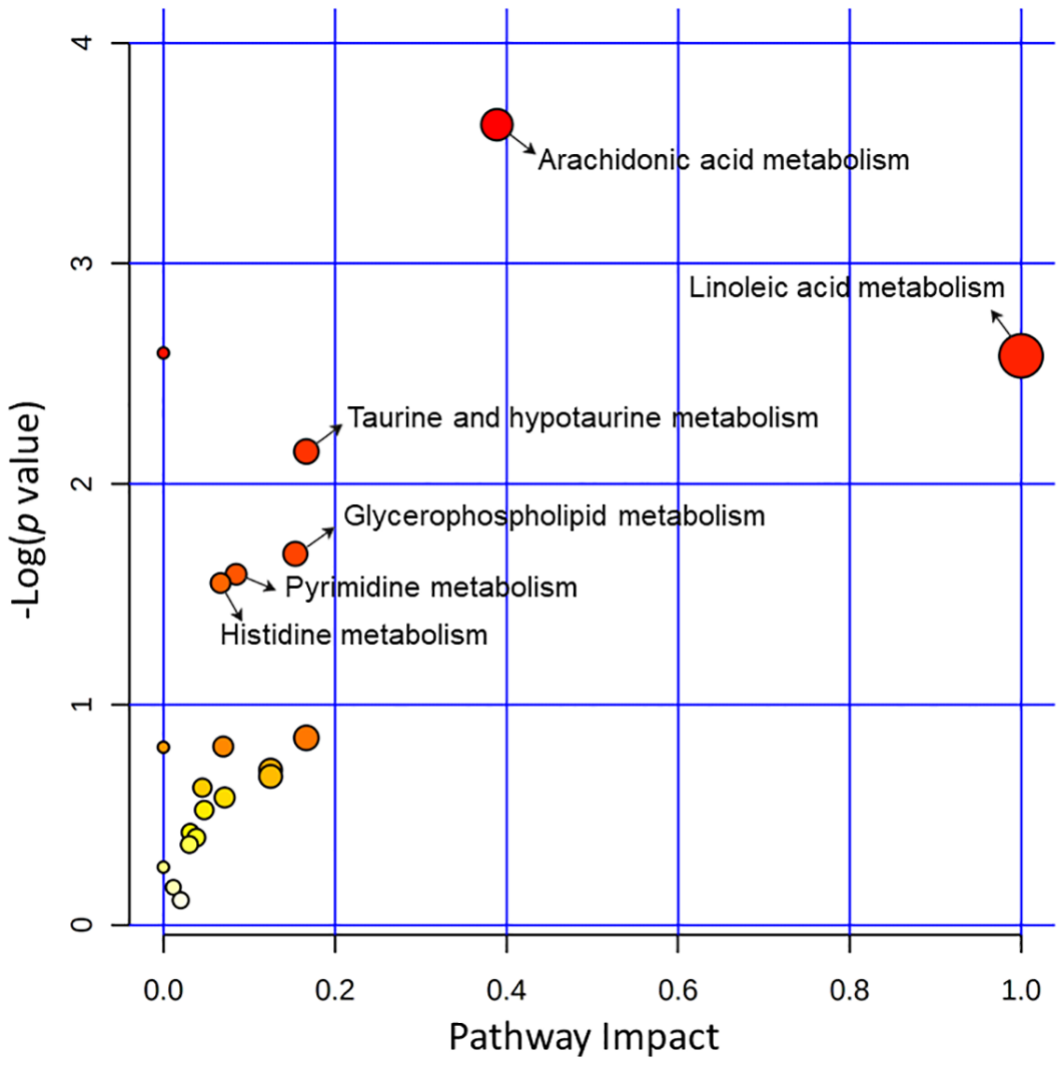
Pathway analysis of the identified differential metabolites between the acutely infected group (AI) and the noninfected group (Ctrl) in ESI− mode and ESI+ mode. Pathway impacts resulting from the differential metabolites were obtained using MetaboAnalyst 5.0. The magnitude of the enrichment ratio was computed as observed hits/expected hits. Pathways with *p* < 0.05 were considered significant.

More than 20 metabolic pathways were influenced in the acutely infected group. Six pathways (linoleic acid metabolism, arachidonic acid metabolism, taurine and hypotaurine metabolism, glycerophospholipid metabolism, pyrimidine metabolism, and histidine metabolism) showed the greatest impact and the lowest *p* values. The three pathways that were most affected were linoleic acid metabolism, arachidonic acid metabolism, and taurine and hypotaurine metabolism (**Figure 5**).

### Time courses of significant perturbations of metabolites

**Figure 6:**
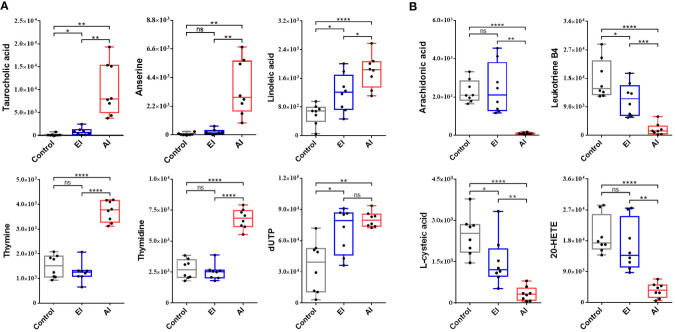
Box and whisker plots showing significantly perturbed metabolites correlated with the dysregulated metabolic pathways. **(A)** Top 6 upregulated metabolites in the enriched pathway. **(B)** Top 4 downregulated metabolites in the enriched pathway. EI, early infected group; AI, acutely infected group; Ctrl, noninfected group. Statistically significant differences in metabolites between groups were determined by t tests (*, *p <*0.05; **, *p <*0.01; ***, *p <*0.001; ****, *p <*0.0001).

A total of ten metabolites that were significantly perturbed were identified and correlated with dysregulated metabolic pathways. Taurocholic acid and linoleic acid levels increased during early infection, and the differences in the levels of these metabolites increased significantly during acute infection (**Figure 6A**). At the beginning of the infection, no abnormalities in anserine, thymine, or thymidine levels were detected, but a significant rise in the levels of these metabolites was observed during acute infection (**Figure 6A**). The dUTP level increased significantly beginning early in the infection and remained constant until the acute infection stage (**Figure 6A**). No abnormalities in arachidonic acid or 20-HETE levels were detected during early infection, but the levels of these metabolites decreased significantly during acute infection (**Figure 6B**). Leukotriene B4 and L-cysteic acid levels were reduced beginning early in the infection, and the change became significant during acute infection (**Figure 6B**). Interestingly, at the acute stage of *B. microti* infection, taurocholic acid and anserine production was upregulated; the levels of these metabolites increased by 69.24-fold and 57.06-fold, respectively, while the arachidonic acid level was downregulated to 0.04 of the control level (**Table 2**).

**Table 2:** Identified perturbed metabolites correlated to the significantly dysregulated metabolic pathways.

Type	Mode	Metabolite	Pathway	VIP	FC	Log_2_FC	*p* value
Up	ESI+	Taurocholic acid	Taurine and hypotaurine metabolism	1.79	69.24	6.11	1.71E-03
	ESI+	Anserine	Histidine metabolism	1.85	57.06	5.83	2.17E-03
	ESI+	Linoleic acid	Linoleic acid metabolism	1.38	2.97	1.57	5.69E-05
	ESI+	Thymine	Pyrimidine metabolism	1.78	2.50	1.32	8.19E-08
	ESI−	Thymidine	Pyrimidine metabolism	1.66	2.48	1.31	6.43E-08
	ESI+	dUTP	Pyrimidine metabolism	1.25	2.30	1.20	1.01E-03
Down	ESI+	Arachidonic acid	Arachidonic acid metabolism	1.93	0.04	-4.52	1.34E-05
	ESI−	Leukotriene B4	Arachidonic acid metabolism	1.58	0.11	-3.24	9.60E-05
	ESI−	L-cysteic acid	Taurine and hypotaurine metabolism	1.57	0.13	-2.90	3.40E-05
	ESI+	20-HETE	Arachidonic acid metabolism	1.68	0.18	-2.46	3.33E-05

VIP, variable importance in projection; FC, Fold change.

## Discussion

*Babesia* is a tick-borne protozoan parasite that infects mammals and has great veterinary, economic and medical impact worldwide (Homer et al., 2000). Human babesiosis, which is also transmitted through blood transfusion, is becoming a global public health concern (Kumar et al., 2021). The clinical symptoms of *Babesia* infection typically include fever accompanied by malaise and fatigue, followed by anemia, anorexia, emaciation, hemoglobinuria and vomiting. In severe cases, death may occur during the acute hemolytic phase of the infection (Akel and Mobarakai, 2017). However, knowledge of the pathophysiology of human babesiosis is still limited. As a result, there is an urgent need for the identification of new biomarkers that can be used to reliably detect *Babesia* infection in patients.

Several “omics” techniques, such as genomics, proteomics, and transcriptomics, are used to observe host−pathogen interactions (Jean Beltran et al., 2017). Untargeted metabolomics is a powerful approach for investigating host-parasite interactions at the biochemical level and discovering new biomarkers of infection (Chienwichai et al., 2022). LC−MS is commonly used to analyze metabolites and can be used to screen for variations in small-molecule metabolites in complex biological samples. Compared with proteomics and transcriptomics, metabolomics focuses on downstream changes resulting from genetic modification or alterations in the external environment; it shows the closest correlation with phenotype and is the ultimate manifestation of gene function (Marchev et al., 2021).

There are only a few existing studies of the metabolome of *Babesia*. Previous investigations assessed differences in the metabolism of free merozoites and infected RBCs (iRBCs) during *B. divergens* infection and in the urine metabolic profiles of dogs with *B. canis* infection using metabolomics approaches (Fernández-Garcia et al., 2021; Kuleš et al., 2021; Beri et al., 2023). Recent research on metabolic profiling was based on erythrocytes separated from *B. microti*-infected mice and found sabotage of metabolic pathways in iRBCs, including amino acid, lipid, and nucleotide metabolism and the tricarboxylic acid cycle (Gong et al., 2023). However, there is still a gap in our knowledge of how the host metabolome changes during *B. microti* infection.

Serum metabolomics, as an important indicator of pathological and physiological state, may contribute to understanding the mechanisms through which diseases occur and progress at the metabolic level and will provide information that facilitates the identification of differential metabolic markers of disease (Zhang et al., 2012). As *Babesia* is a blood parasite, serum metabolomics directly reflects the infection-related metabolic changes in its host (Antunes et al., 2017). The only study that has been conducted to date on the serum metabolomics of *Babesia* is a study of dogs with *B. canis* infection; in that study, differentially abundant metabolites were found that indicated the involvement of various pathways during infection. The involved pathways included phenylalanine, tyrosine, and tryptophan biosynthesis and alanine, aspartate, glutamate, cysteine and methionine metabolism (Rubić et al., 2022).

In the current study, an untargeted LC−MS-based method was used to identify different metabolic signatures and perturbed metabolites in the serum of mice during the early and acute stages of infection with *B. microti*. Taurocholic acid is a bile acid and is the product of the conjugation of cholic acid with taurine. Like other bile acids, taurocholic acid acts as a detergent to solubilize fats for absorption, but it is not itself absorbed by the small intestine (Ridlon et al., 2016). Generally, high serum levels of taurocholic acid are associated with biliary damage and liver injury (Tian et al., 2021). As taurocholic acid is a nonessential amino acid, the observed 69.24-fold upregulation of taurocholic acid in the serum of infected mice supports the idea that *Babesia* infection induces hepatocellular injury in the host, as our previous study demonstrated (Liu et al., 2021). Anserine is a derivative of carnosine. Unlike carnosine, which is rapidly cleaved by carnosinase, the anserine in serum is methylated and is therefore more stable and resistant to degradation (Katakura et al., 2017). Considering that anserine is a potent antioxidant that reduces the oxidative stress caused by *B. microti* infection and brings the organism towards redox homeostasis (Peters et al., 2018), the observed 57.06-fold increase in the anserine level in serum may be a result of the host’s defense against parasite invasion. Polyunsaturated fatty acids (PUFAs) kill microbes through their direct action on microbial cell membranes. PUFAs enhance the generation of free radicals and augment the formation of lipid peroxides that are cytotoxic to microbes. PUFAs also increase the formation of bioactive metabolites such as lipoxins, maresins, prostaglandins, protectins and resolvins, all of which are compounds that enhance the phagocytic action of macrophages and leukocytes (Das, 2018). Arachidonic acid, a polyunsaturated fatty acid, has been reported to be an endogenous antiparasitic and an agent that modulates the immune response to malaria and schistosomiasis (Kilunga Kubata et al., 1998; Tallima et al., 2020). Thus, the rapid growth of parasites in the mice in our study led to the depletion of arachidonic acid, and this was manifested as a 0.04-fold downregulation in the level of arachidonic acid during the acute stage of *B. microti* infection.

In conclusion, our study provides novel insights into the pathogenesis of *B. microti* within its host and improves the current understanding of the biology of *B. microti* through LC−MS metabolomics analysis. The findings of this study indicate that taurocholic acid, anserine, and arachidonic acid show potential as candidate serological biomarkers for *B. microti* infection and contribute to the discovery of further effective anti-*B. microti* therapeutics. Further validation, including evaluation of the specificity, sensitivity, and clinical utility, is needed to establish the reliability and clinical relevance of above candidate metabolites as biomarkers.

## Data availability statement

The datasets presented in this study can be found in online repositories. The names of the repository/repositories and accession number(s) can be found below: The metabolomics data are available in the MetaboLights database (MTBLS7399).

## Ethics statement

The animal study was reviewed and approved by the Science Ethics Committee of the Hubei University of Arts and Science (Permit No. 2021-012).

## Author contributions

JX and ML designed the study. LS, CW, RW, and XH carried out the experiments. SL, WL, and AD performed the statistical analysis. LS, CW, RW, JX, and ML drafted the manuscript. SJ, EG, HL, and XX revised the manuscript. All authors contributed to the article and approved the submitted version.

## Supplementary material

Supplementary Figure 1 Orthogonal partial least squares discriminant analysis (OPLS-DA) of the metabolite profile. (A) and (C): OPLS-DA score plots between the early infected group (EI) and the noninfected group (Ctrl) in ESI− mode (A) and ESI+ mode (C). (B) and (D): OPLS-DA model with 200 permutation tests between EI and Ctrl in ESI− mode (B) and in ESI+ mode (D).

Supplementary Figure 2 Orthogonal partial least squares discriminant analysis (OPLS-DA) of the metabolite profile. (A) and (C): OPLS-DA score plots between the early infected group (EI) and the acutely infected group (AI) in ESI− mode (A) and ESI+ mode (C). (B) and (D): OPLS-DA model with 200 permutation tests between EI and AI in ESI− mode (B) and in ESI+ mode (D).
