# Supplementary figures and images for: Serum metabolomic profiles in BALB/c mice induced by Babesia microti infection
Source: Front Cell Infect Microbiol. 2023 Apr 28;13:1179967. doi: 10.3389/fcimb.2023.1179967 (PMC10176453; doi:10.3389/fcimb.2023.1179967)

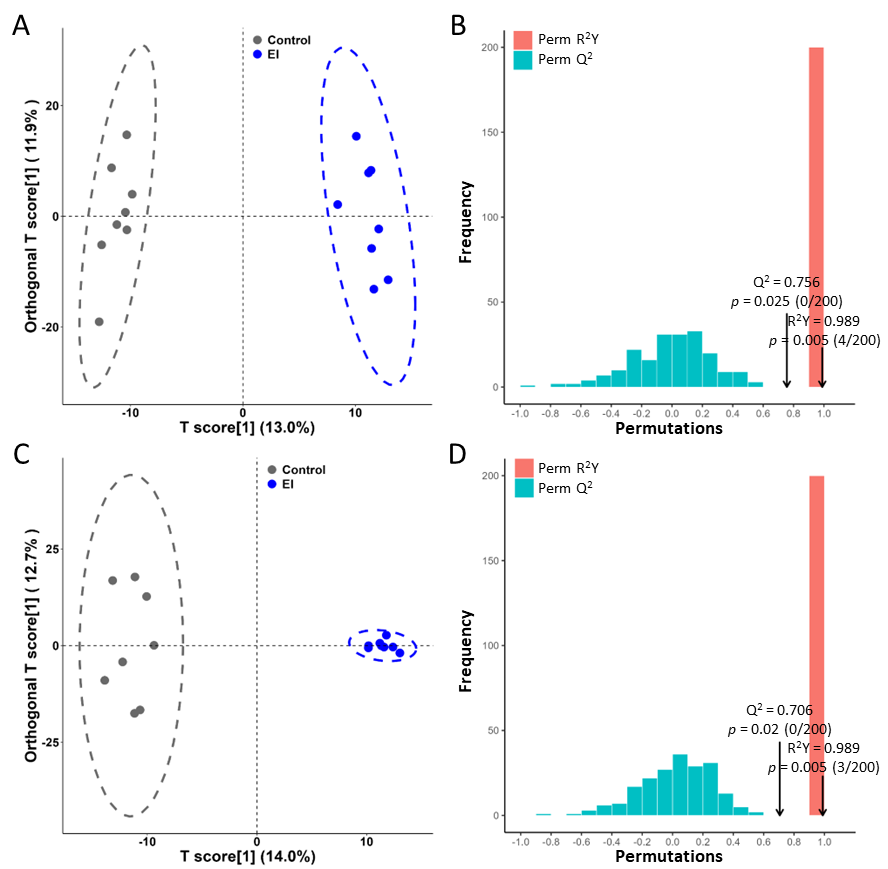

Supplement: Supplementary Figure 1 — Orthogonal partial least squares discriminant analysis (OPLS-DA) of the metabolite profile. (A) and (C): OPLS-DA score plots between the early infected group (EI) and the noninfected group (Ctrl) in ESI− mode (A) and ESI+ mode (C). (B) and (D): OPLS-DA model with 200 permutation tests between EI and Ctrl in ESI− mode (B) and in ESI+ mode (D). [file Image_1.tif]

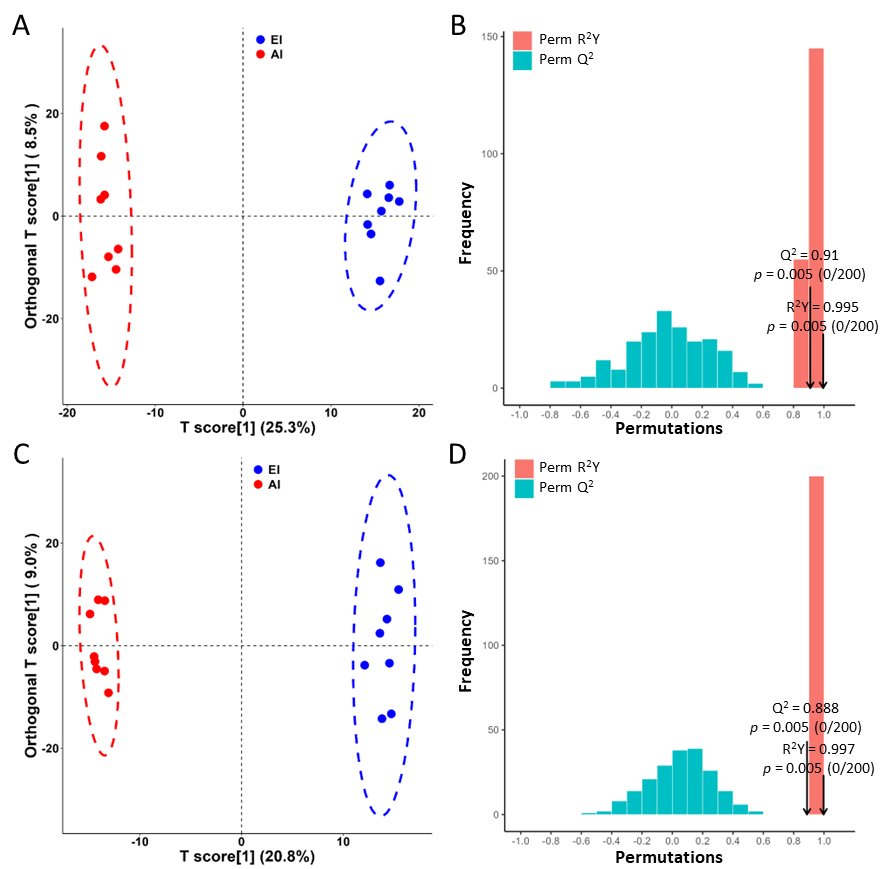

Supplement: Supplementary Figure 2 — Orthogonal partial least squares discriminant analysis (OPLS-DA) of the metabolite profile. (A) and (C): OPLS-DA score plots between the early infected group (EI) and the acutely infected group (AI) in ESI− mode (A) and ESI+ mode (C). (B) and (D): OPLS-DA model with 200 permutation tests between EI and AI in ESI− mode (B) and in ESI+ mode (D). [file Image_2.tif]
